# Differences of microparticle patterns between sickle cell anemia and hemoglobin SC patients

**DOI:** 10.1371/journal.pone.0177397

**Published:** 2017-05-10

**Authors:** Yohann Garnier, Séverine Ferdinand, Maryse Etienne-Julan, Gisèle Elana, Marie Petras, Lydia Doumdo, Benoit Tressières, Marie-Laure Lalanne-Mistrih, Marie-Dominique Hardy-Dessources, Philippe Connes, Marc Romana

**Affiliations:** 1 Unité Biologie Intégrée du Globule Rouge, Université des Antilles, Inserm 1134, laboratoire d’Excellence GR-Ex, Paris, France; 2 Unité Transversale de la Drépanocytose, CHU de Pointe-à-Pitre, Pointe-à-Pitre, Guadeloupe, France; 3 Pôle mère-enfant, CHU de Fort de France, Fort de France, Martinique; 4 Centre d’Investigation Clinique Antilles Guyane, Inserm/DGOS CIC 1424, Pointe-à-Pitre, Guadeloupe, France; 5 Institut Universitaire de France, Paris, France; 6 Laboratoire LIBM EA7424, Equipe « Biologie Vasculaire et du Globule Rouge », laboratoire d’Excellence GR-Ex, Université Claude Bernard Lyon 1, Villeurbanne, France; Universidade de Sao Paulo, BRAZIL

## Abstract

Sickle cell anemia (SCA) and hemoglobin SC (HbSC) disease are the two most common forms of sickle cell disease (SCD), a frequent hemoglobinopathy which exhibits a highly variable clinical course. Although high levels of microparticles (MPs) have been consistently reported in SCA and evidence of their harmful impact on the SCA complication occurrences have been provided, no data on MP pattern in HbSC patients has been reported so far. In this study, we determined and compared the MP patterns of 84 HbSC and 96 SCA children, all at steady-state, using flow cytometry. Most of circulating MPs were derived from platelets (PLTs) and red blood cells (RBCs) in the two SCD syndromes. Moreover, we showed that HbSC patients exhibited lower blood concentration of total MPs compared to SCA patients, resulting mainly from a decrease of MP levels originated from RBCs and to a lesser extent from PLTs. We did not detect any association between blood MP concentrations and the occurrence of painful vaso-occlusive crises, acute chest syndrome and pulmonary hypertension in both patient groups. We also demonstrated for the first time, that whatever the considered genotype, RBC-derived MPs exhibited higher externalized phosphatidylserine level and were larger than PLT-derived MPs.

## Introduction

Sickle cell disease (SCD) is a group of genetic disorders having in common the production of the abnormal hemoglobin S (HbS) instead of hemoglobin A. Sickle cell anemia (SCA), *i*.*e*. the homozygous form of SCD, results from a single base mutation in exon 1 of the β-globin gene which causes the substitution of valine for glutamic acid at the sixth position of the β-globin chain. When deoxygenated, HbS polymerizes and induces the sickling of red blood cells (RBCs), leading to decreased deformability and increased fragility of these cells. Sickle RBCs do not easily flow through the microcirculation, causing frequent vaso-occlusive episodes, and exhibit a reduced life-span, responsible for the anemic status of affected patients. Recurrent HbS polymerization induces numerous RBC and systemic pathophysiological abnormalities associated with clinical manifestations such as vaso-occlusive crises, acute chest syndrome and multi-organ disease [1]. Co-inheritance of HbS and HbC, another abnormal Hb, is at the origin of the second most frequent sickle cell syndrome, *i*.*e*. hemoglobin SC disease. HbC is the outcome of a single amino acid change at the same position in β-globin chain but with a lysine replacing glutamic acid. The tendency of HbC to crystallize is enhanced in HbSC patients, which promotes RBC dehydration further facilitating HbS polymerization and reducing RBC deformability [2–3]. Hematologically, HbSC disease is distinct from SCA, with higher hemoglobin (Hb) levels, lower rates of hemolysis and lower white blood cell counts [4]. HbSC disease is usually considered as a mild form of SCD but recent studies reported higher prevalence of several complications such as retinopathy and osteonecrosis in HbSC than in SCA patients [5,6]. In addition, the rate of hospitalized vaso-occlusive events in HbSC, particularly in children, is non negligible [7]. Although the molecular and cellular mechanisms at the origin of the polymerization of hemoglobin S and crystallization of hemoglobin C are now well described [8,9], the wide clinical variability of SCD remains poorly understood [10].

Like in several cardiovascular and metabolic diseases [11], high plasma concentration of microparticles (MPs) has been consistently reported in SCA patients at steady-state [12–18], with a further rise during vaso-occlusive crisis (VOC) episodes [17–19]. These extracellular vesicles are membrane-derived vesicles smaller than 1 μm that are shed from any cell type in response to cell activation, cell stress or apoptosis [20]. Composed of a phospholipid bilayer and enclosing cytosolic components such as enzymes and mRNA derived from their parental cells, MPs are involved in the cellular cross-talk of several physiological and pathophysiological pathways. Indeed, the functional consequences of MPs have been documented in several disorders or clinical conditions such as myocardial infarction, preeclampsia, neovascularization, metastasis, thrombosis and inflammation [21]. In the context of cardiovascular disorders and transfusion, these MPs may promote inflammation and cell proliferation and may interfere with normal vascular responses [22–23].

The involvement of these sub-cellular elements in several pathophysiological processes of SCD has been strongly suggested. Indeed, it has been shown that MPs originated from sickle RBCs may trigger coagulation, increase the production the production of radical oxygen species by endothelial cells, induce erythrocyte adhesion to endothelial cells and endothelial cell apopotosis, as well as, trigger renal vaso-occlusive crisis in a sickle mouse model [24–26]. Furthermore, several studies also supported a role of MPs in the occurrence of several complications in patients with SCA [15,27–28]. While these data strongly suggest that MPs are not only bio-markers but also bio-effectors in SCA, no study looked at the presence and/or at the circulating MP profile in HbSC patients.

This study was therefore devoted to determine and compare quantitatively and qualitatively MP patterns between 84 HbSC and 96 SCA children. In addition, to gain insight into the role of MPs in HbSC disease as well as in SCA, we analyzed the associations between MP levels and several markers of SCD clinical severity.

## Materials and methods

### Patients

The study included 180 consecutive SCD children regularly followed-up by two sickle cell centers based in the French Caribbean islands and identified by new-born screening programs: 109 (59 SCA and 50 HbSC) children followed-up in Guadeloupe and 71 (37 SCA and 34 HbSC) children in Martinique. Overall, 91 boys and 89 girls between 8 and 16 years old were included. Twenty-nine SCA children were under hydroxycarbamide (HC) treatment for more than 6 months, with an average dose of 20.7±2.8 mg/kg per day. No HbSC children was under HC therapy. All children were at steady state at inclusion, i.e. no blood transfusions in the previous three months, absence of acute episodes (infection, VOC, acute chest syndrome (ACS), stroke and priapism) for at least one month before enrolment. The study was conducted in accordance with the Declaration of Helsinki and was approved by the Regional Ethics Committee (CPP Sud/Ouest Outre Mer III, Bordeaux, France; registration number 2009-A00211-56). Children and their parents were informed of the purpose and procedures of the study, and gave written consent.

### Clinical data

Charts were retrospectively reviewed by 3 physicians to recognize all ACS and VOC episodes from birth to the time of blood sampling, based on previously described criteria [7]. The rates of VOC were calculated for each child by dividing the total number of painful VOC episodes by the number of patient-years [7] and two subgroups were constituted according to the median VOC rate. The tricuspid regurgitant jet velocity (TRJV), available for 102 children at steady-state, was also recorded. The Philips IE33 system (Philips Medical Systems, Bothell, WA) was used for evaluating pulmonary hypertension according to the criteria of the American Society of Echocardiography [29]. A TRJV ≥ 2.5 m/sec was considered abnormal [30–31].

### Laboratory methods

The SCD diagnosis for these patients has been previously established at the referent laboratories of the university hospitals of Pointe-à-Pitre (Guadeloupe) and Fort de France (Martinique) by isolectrofocusing electrophoresis (Multiphor II^™^ System, GE HEALTH CARE, Buck, UK) and high performance liquid chromatography (VARIANT^™^, Bio Rad Laboratories, Hercules, CA, USA). In addition to Hb analysis, SCA diagnosis was confirmed by DNA analysis as previously described [32]. Blood count analysis including reticulocyte (RET) counts was performed using a hematology analyzer (Max M-Retic, Coulter, USA). Serum lactate dehydrogenase (LDH), aspartate aminotransferase (ASAT), total bilirubin (BIL) and unconjugated bilirubin (UNBIL) levels were determined using standard methodologies.

### Isolation of MPs and flow cytometry analysis

**Fig 1:**
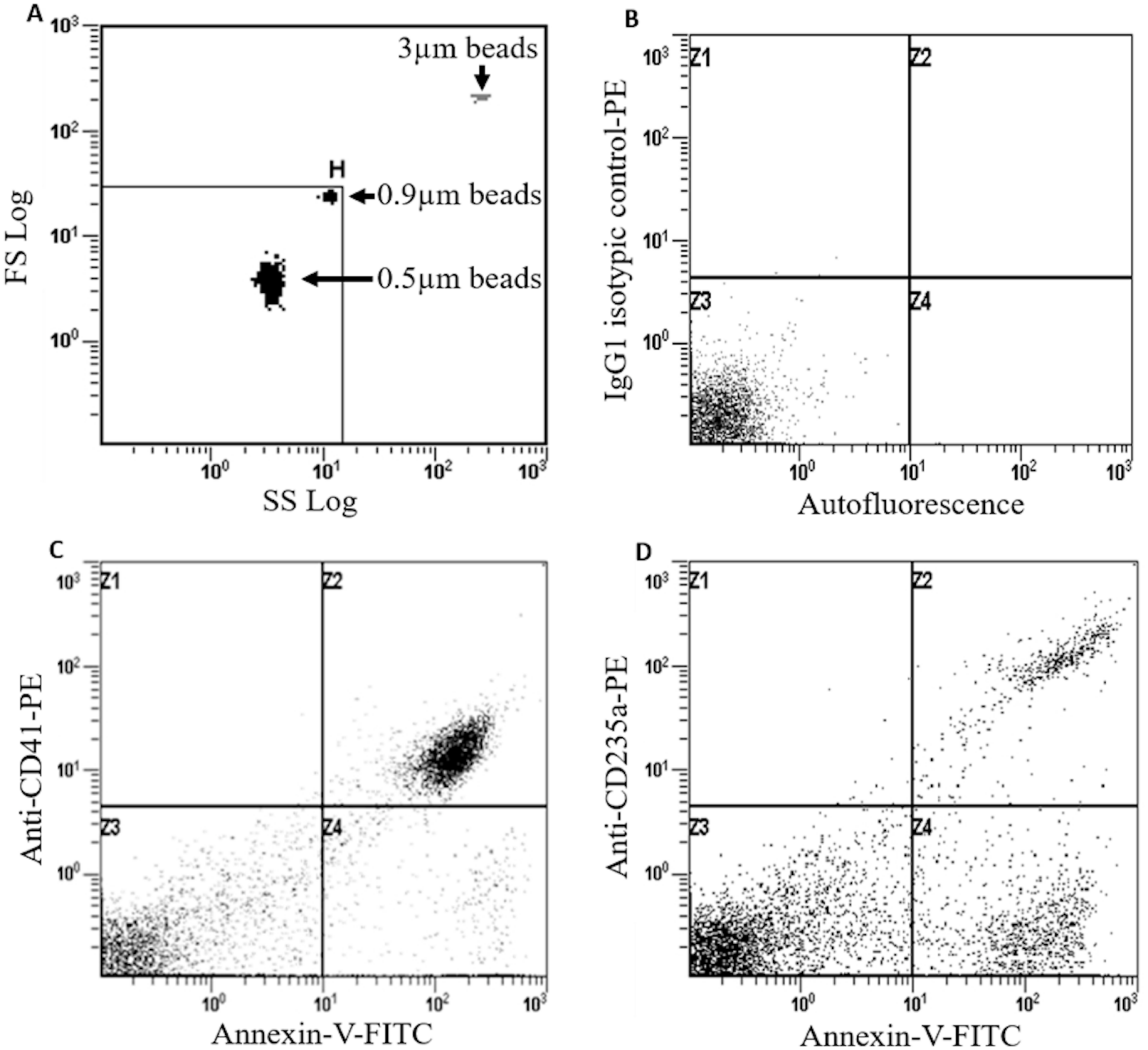
MP characterization by flow cytometry. **A**: Acquisition gate (H) was based on forward- and side-scatter values of 0.9 mm-large calibration beads; **B**: autofluorescence was determined using isotopic control (IgG-PE); **C**: platelet-derived MPs or **D**: erythrocyte-derived MPs were labelled with FITC-annexin-V (FL1) and PE-conjugated monoclonal antibodies directed against CD41 or CD235a, respectively.

MPs were isolated and analyzed as previously reported, using a FC500 flow cytometer (Beckman Coulter, FL USA) [16]. Briefly, platelet-poor plasma, obtained from blood collected on 3.2% trisodium-citrate tube after centrifugation (1,500g, 10 min, room temperature), was submitted to ultracentrifugation (20,000g, 20 min, room temperature) to allow extraction of MPs. The pellet was subsequently washed twice in working buffer (WB): (10 mM HEPES pH 7.4, 136 mM NaCl, 5 mM KCl, 2 mM MgCl_2_) containing 5 mM of EDTA for the first washing step, or no EDTA for the second one. WB was added to the pellet for resuspension, and this MPs solution, prepared either in Guadeloupe or in Martinique, was stored at - 80°C in the biological resource centers of Guadeloupe or Martinique. Flow cytometry analysis was performed in Guadeloupe. Fluoresceinisothiocyanate (FITC)-conjugated annexin-V (Beckman Coulter) and phycoerythrin (PE)-coupled cell type-specific monoclonal antibodies (MoAbs) were incubated with extracted MPs, thereby allowing the determination of MPs cell type-of-origin. The cell type markers-specific MoAbs were: anti-CD14 (GPI, clone M5E2, IgG2a; for monocytes), anti-CD15 (Lewis X, clone HI98, IgM; for granulocytes), anti-CD41 (GPIIb, clone HI98, IgG1; for platelets), anti-CD106 (VCAM1, clone 51–10 C9, IgG1; for endothelial cells), anti-CD235a (Glycophorin A, clone 11E4B-7-6, IgG1; for erythrocytes). IgG1 (679.1Mc7), IgG2a (7T4-1F5) or IgM (G20-127) were used as isotypic controls. All MoAbs were from Beckman Coulter. The acquisition gate for MPs was standardized using the megamix kit, a blend of size-calibrated fluorescent microbeads (0.5, 0.9 and 3 μm; Biocytex, Marseille, France) according to the supplier’s instructions. MPs were defined as elements smaller than 1μm positively labeled with annexin-V. This analysis procedure and the flow cytometer used allowed the detection and analysis of sub-cellular elements of diameters ranging from 400 to 900 nm, known to be composed of MPs mainly [33]. However, some of these sub-cellular elements detected could also be apoptotic bodies, another type of extracellular vesicles, since their diameters overlap with those of MPs [34]. Flow-Count^™^ fluorospheres (Beckman Coulter) were used to obtain absolute MPs quantification. Flow cytometry analysis of MPs is represented in Fig 1. Blood MP concentrations were calculated from plasma MP concentrations corrected by the hematocrit values.

### Statistical analysis

Quantitative variables were summarized as median with the interquartile range (IQR) and categorical data were expressed in percentage. Intergroup differences were analyzed by Mann-Whitney non-parametric test or Student t test when appropriate. Categorized variables were analyzed using Fisher’s exact test. Bivariate correlations were tested by Spearman’s rank correlation. The characteristics of MPs originated from RBCs and PLTs were compared in each SCD syndrome as well as the characteristics of each cell type specific MPs between the two SCD syndromes using Mann-Whitney test. To identify factors independently associated with RBC and PLT-derived MP concentrations, multivariate linear regression analyses were performed with the inclusion of clinical and laboratory variables that were found to be associated with MP concentrations in univariate analyses. Step-by-step downward iterations were done to retain the independent parameters. The model analyses were performed using log-transformed MP concentrations to normalize the data distribution. The significance level was defined as *p <* 0.05. Analyses were conducted using SPSS (v. 21, IBM SPSS Statistics, Chicago, IL) and GraphPad Prism (v.7, GraphPad, La Jolla, CA).

## Results

The hematological, biochemical and clinical features of the 180 SCD patients included in the present study are shown in Table 1. As expected, HbSC children were less anemic and they exhibited lower fetal hemoglobin (HbF), hemolytic markers levels, white blood cell (WBC) and platelet (PLT) counts than SCA children. Fewer children with a past-history of VOC or ACS were also detected in the HbSC children group, whereas no difference was observed for abnormal TRJV between the two groups.

**Table 1:** Biological and clinical characteristics of the SCD studied patients.

	SCA patients	HbSC patients	*p* values
n	96	84	-
Sex ratio (M/F)	47/49	44/40	0.65^&^
Age (years)	11.24 ± 2.4	11.54 ± 2.4	0.40*
Hb (g/dL)	7.9 (7.1–8.7)	11.3 (10.6–11.9)	**<10**^**−3#**^
HbF (%)	7.6 (4.3–12.2)	1.9 (1.2–3.2)	**<10**^**−3#**^
RET (10^9^/L)	269 (200–342)	116 (96.5–153)	**<10**^**−3#**^
LDH (IU/L)	588 (466–700)	292 (264–333)	**<10**^**−3#**^
Total bilirubin (μM)	47 (35–68)	21 (16–30)	**<10**^**−3#**^
Unconjugated Bilirubin (μM)	37 (25–59)	17 (12–23)	**<10**^**−3#**^
ASAT (IU/L)	49 (37–52)	26 (21–29)	**<10**^**−3#**^
WBC (10^9^/L)	10.8 (8.7–12.6)	5.9 (4.7–8.2)	**<10**^**−3#**^
PLT (10^9^/L)	442 (364–495)	212 (168–316)	**<10**^**−3#**^
HC treatment	29	0	-
History of VOC (yes/no)	82/14	57/27	**0.007**^&^
VOC rate	0.33 (0.11–0.78)	0.14 (0–0.31)	**0.0004**^**#**^
History of ACS (yes/no)	48/48	15/69	**<10**^**−3**&^
TRJV ≥ 2.5m/s (yes/no)	14/46	5/37	0.19^&^

Quantitative variables were summarized as means ± standard deviation or as the median with the interquartile range (IQR) according to their distributions. Intergroup differences were assessed using unpaired t test (*), Mann Whitney test (#) or chi 2 test (&). Significant *p* values are in bold. Hb: hemoglobin; HbF: fetal hemoglobin; RET: reticulocytes; LDH: lactate dehydrogenase; ASAT: aspartate aminotransferase, WBC: white blood cell; PLT: platelet; TRJV: tricuspid regurgitation jet velocity; VOC: vaso-occlusive crisis; ACS: acute chest syndrome; HC: hydroxycarbamide.

Compared to SCA children, those affected by HbSC disease exhibited a significantly lower concentration of total MPs, resulting mainly from a decrease of MPs originated from RBCs and to a lesser extent from PLTs (Table 2).

**Table 2:** Cellular origins and blood MP concentrations of SCA and HbSC patients.

	SCA patients	HbSC patients	*p* values
Total MPs (MPs/μl)	8,507 (4,705–18,022)	4,587 (2,605–10,915)	**0.001**
RBC-MPs (MPs/μl)	631 (272–1,498)	260 (151–540)	**<10**^**−3**^
PLT-MPs (MPs/μl)	6,485 (3,310–15,537)	4,014 (2,154–9,570)	**0.008**
Mono-MPs (MPs/μl)	31 (0–111)	19 (4–61)	0.194
Neutro-MPs (MPs/μl)	24 (1–103)	16 (0–62)	0.272
Endo-MPs (MPs/μl)	24 (1–107)	16 (2–60)	0.440

MP concentrations were expressed as median with the interquartile range. Significant *p* values are in bold. Mono: monocyte; Neutro: neutrophil; Endo: endothelial.

We also detected lower concentrations of total MP, RBC- and PLT-derived MP in HbSC patients compared to SCA patients not treated by HC (4,587 (IQR 2,605–10,915) vs 12,155 MP/μl (IQR 5,916–19,510), *p* < 0.001; 260 (IQR 151–540) vs 755 MP/μl (IQR 401–1,782), *p* < 0.001; 4,014 (IQR 2,154–9,570) vs 8,643 MP/μl (IQR 4,383–15,697), *p* < 0.01, respectively) while no difference was detected between HbSC and HC-treated SCA patient (doi.org/10.6084/m9.figshare.4955201.v1).

**Fig 2:**
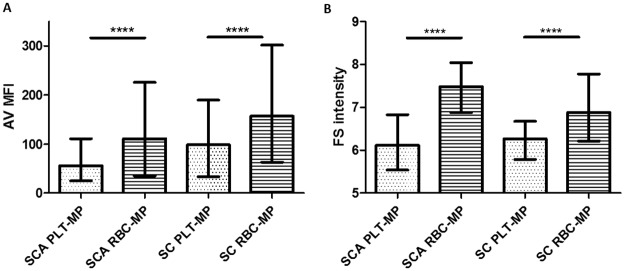
Comparison of RBC- and PLT-derived MP characteristics between SCA and HbSC patients. **A**: mean fluorescence intensity of annexin V; **B**: mean forward scatter index. The value distributions are represented as box and whiskers (min to max). ****: p < 10^−4^.

The mean fluorescence intensity (MFI) of Annexin V-FITC, a parameter reflecting the density of phosphatidylserine (PS) on the MPs membrane outer leaflet, and the mean forward scatter (FS) index, a parameter correlated with MPs size, were compared between SCA and HbSC children. For this analysis, we considered the two most common blood cell type-specific MPs detected, *i*.*e*. MPs released by RBCs and PLTs. As illustrated in Fig 2A, we observed higher MFI values for RBC-derived MPs than for PLT-derived MPs in the two SCD groups. In both groups, we also detected higher mean FS indexes for MPs originated from RBCs (Fig 2B and S1 Fig). Moreover, RBC- and PLT-derived MPs isolated from HbSC children exhibited higher MFI than those isolated from SCA children (*p* = 0.009 and *p* = 0.002 respectively). No mean FS index difference was detected between the two groups for PLT-derived MPs (*p* = 0.5) while higher FS values for RBC-derived MPs were detected in SCA patients compared to HbSC patients (*p* = 0.004).

We then compared MP patterns between children having a positive history of VOC or ACS or those with TRJV ≥ 2.5 m/sec with those who did not experience these conditions in each SCD group. Since HC treatment modifies the hematological parameters and the clinical expression of the disease (S1 Table), and is associated with a decrease of MP concentration (S2 Table), SCA children treated with HC were excluded from this analysis.

**Fig 3:**
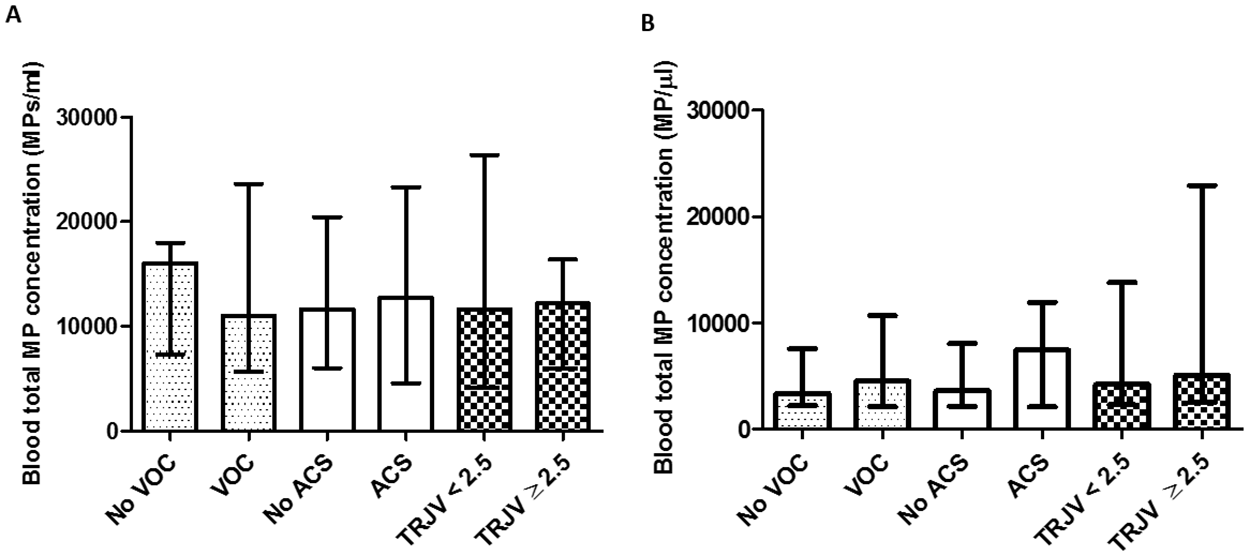
Comparison of total MP concentrations between not HC-treated SCA and HbSC patients classified according to the occurrence of VOC, ACS and abnormal TRJV. **A**: Not HC-treated SCA children classified according to VOC, ACS and TRJV ≥ 2.5 m/s occurrences respectively. **B**: HbSC children classified according to VOC ACS and TRJV ≥ 2.5 m/s occurrences respectively. Blood total MP concentrations are represented as median with interquartile range.

No significant difference in total blood MP concentrations was observed between children with and without one of the complication investigated (Fig 3). Similar results were obtained for RBC- and PLT-derived MPs (doi.org/10.6084/m9.figshare.4955216.v1, doi.org/10.6084/m9.figshare.4955225.v1).

The relationships between the concentrations of RBC- and PLT-derived MPs, anemia and hemolytic markers are shown in Table 3. For RBC-derived MPs, we detected an inverse correlation with the level of hemoglobin (Hb) and HbF, and positive relationships with RET count, UNBIL, LDH and ASAT levels in SCA patients not treated by HC. A positive correlation was observed between RBC-derived MPs and RET counts in HbSC patients. For PLT-derived MPs, relationships with Hb and UNBIL levels were observed in SCA patients without HC treatment, while correlations with RET count and ASAT level were detected in the HbSC group.

**Table 3:** Correlation between MP concentrations and markers of hemolysis/anemia in SCD patients.

	SCA patients without HC treatment			HbSC patients		
	Spearman ρ	95% CI	*p* values	Spearman ρ	95% CI	*p* values
RBC-derived MP						
Hb	-0.33	-0.51 to -0.12	**0.002**	-0.09	-0.31 to 0.13	0.41
HbF	-0.34	-0.54 to -0.11	**0.004**	-0.03	-0.28 to 0.21	0.78
RET	0.34	0.13 to 0.53	**0.0015**	0.31	0.08 to 0.49	**0.009**
UNBIL	0.33	0.11 to 0.52	**0.004**	0.04	-0.20 to 0.27	0.74
LDH	0.38	0.12 to 0.58	**0.004**	-0.12	-0.37 to 0.14	0.35
ASAT	0.31	0.09 to 0.50	**0.005**	-0.01	-0.25 to 0.23	0.93
PLT-derived MP						
Hb	-0.22	-0.43 to -0.004	**0.04**	-0.2	-0.40 to 0.03	0.08
HbF	-0.21	-0.43 to 0.04	0.09	0.01	-0.23 to 0.26	0.92
RET	0.15	-0.08 to 0.36	0.18	0.28	0.06 to 0.48	**0.013**
UNBIL	0.24	0.02 to 0.44	**0.03**	-0.19	-0.40 to 0.03	0.08
LDH	0.13	-0.14 to 0.38	0.32	0.21	-0.05 to 0.44	0.10
ASAT	0.04	-0.18 to 0.28	0.67	0.25	0.02 to 0.46	**0.03**

Correlations were estimated by Spearman’s rank correlation (ρ) in SCD patients. CI: confidence interval. Significant *p* values are in bold.

Multivariate linear regression analyses were performed to assess the presence of independent associations between clinical/laboratory variables and RBC- and PLT-derived MP concentrations. RET (beta = 0.25, 95% confident intervals (CI): 0 to 0.002, *p* = 0.014) and LDH (beta = 0.21, 95% CI: 0 to 0.001, *p* = 0.035) remained independently associated with MPs originated from RBCs in SCA children. Hb (beta = -0.25, 95% CI: -0.16 to -0.003, *p* = 0.04) and HC treatment (beta = -2.09, 95% CI: -0.41 to -0.012, *p* = 0.038) were independently associated with PLT-derived MPs in SCA children. RET was the only parameter independently associated with RBC-derived MPs (beta = 0.3, 95% CI: -0.001 to -0.005, *p* = 0.005) or PLT-derived MPs (beta = 0.26, 95% CI: 0.001 to 0.0056, *p* = 0.018) in HbSC children.

## Discussion

In this study, we described the quantitative and qualitative MP pattern in HbSC children and identified several distinct features compared to those of SCA children. Although most of circulating MPs were derived from PLTs and RBCs in the two SCD syndromes studied, we showed that HbSC patients exhibited lower blood concentration of total MPs, RBC- and PLT-derived MPs than SCA patients not treated with HC. Moreover, we also demonstrated for the first time that whatever the considered genotype, RBC-derived MPs exhibited higher externalized PS and were larger than PLT-derived MPs. Finally, we did not detect any association between MPs concentration and the occurrence of three major complications in both patient groups.

The mechanisms leading to MPs shedding from cytoplasmic membrane remain not fully understood and differ from a cell-type to another [20,35]. These processes are described as involving increased cytosolic calcium, re-organization of cytoskeleton proteins, associated with weakening of their association with the lipid bilayer, externalization of PS and imply distinct enzymatic activities according to the cell type. In SCA, several proteins involved in the shedding of MPs by RBCs have been identified, such as thrombospondin-1 [24] or acid sphingomyelinase [36]. Two major pathophysiological mechanisms have been associated with the increased vesiculation of sickle RBCs: 1) repeated RBC sickling/unsikling events [37–38], and 2) oxidation of sickle RBC membrane proteins [39]. These two phenomena are also at the origin of the fragility of sickle RBCs, explaining the marked hemolysis and anemia in SCD [40–41]. Indeed, as previously demonstrated [14–16,42], it was not surprising to observe significant correlations between several hemolytic markers and the level of RBC-derived MP concentration in both SCA and HbSC patients. It is also tempting to speculate that the lower RBC-derived MP concentration detected in HbSC compared to SCA patients was due to lower oxidative stress and limited sickling/unsickling episodes in the former group compared to the latter one. The mechanisms involved in the shedding of MPs from RBCs such as the involvement of thrombospondin-1 and sphingomyelinase or the exacerbation of oxidative stress, not addressed in the present study, clearly deserve further investigations.

Since nearly every component of hemostasis is altered in the direction of a pro-coagulant phenotype in SCD, this disorder has been referred to as a “hypercoagulability state” [43]. In addition to high plasma levels of PLT-released proteins such as thrombospondin-1, platelet factor 4 [44] or sCD40L [45], increased PLT expression of CD62P (P-selectin) and CD40L has been described in SCA patients [46], demonstrating abnormal activation of this blood cell type element. In agreement with previous studies, our study showed that Hb level and RET count were independently associated with the level of PLT-derived MPs in SCA and HbSC patients, respectively, which suggests a link between hemolysis/anemia and platelet activation in SCD [14–15,42].

MPs exhibit pro-coagulant properties through different mechanisms depending on the presence of PS and tissue factor at their surface [12,47–48]. The higher density of externalized PS in RBC-derived MPs and bigger size of RBC-derived MPs compared to PLT-derived MPs could explain why RBC-derived MPs but not PLT-derived MPs seem to have a strong impact on blood coagulation [14,43]. The increase of externalized PS in MPs originated from RBCs compared to those derived from PLTs is consistent with the higher PS level of the cytoplasmic membrane reported in the former than in the latter [49,50]. Unexpectedly, we observed that PLT- and RBC-derived MPs isolated from HbSC patients have higher levels of externalized PS than those isolated from SCA patients. These observations cannot be explained by the size of MPs since no difference was observed between the two groups for PLT-derived MPs and higher FS values were detected for RBC-derived MPs isolated from SCA patients. The higher PS exposure of MPs isolated from HbSC patients seem to have a limited impact on blood coagulation since lower plasma levels of coagulation markers have been detected in this sickle cell syndrome compared to SCA [6], an observation also in agreement with their observed lower blood MPs concentration.

As expected, SCA patients under HC treatment had lower MPs levels than those without HC [16]. Unexpectedly, we did not detect any association between MP levels and occurrences of painful VOC, ACS and TRJV ≥ 2.5 m/s in either SCA or HbSC patients. These results contrast with previous findings where higher MP concentrations were reported in SCA patients with frequent VOC [15,27] or other complications such as ACS or pulmonary hypertension [15]. Differences in study design and/or methods used to analyze MPs may account for these discrepancies. In our previous report, the clinical history of adult SCA patients was recorded over a two years-period [27] compared to the more accurate life-long recording period of the present study. Furthermore, the age difference of the patients included in these two studies may also be involved in the discrepancy observed although no report to our knowledge had documented so far an age-related MP distribution. The design of our study also differed from that of Tantawy et al [15] in several issues. Indeed, they have studied young children and adolescents with various sickle cell syndromes (SCA and Sβ-thalassemia) with different clinical status (at steady-state and during crisis). Furthermore the pre-analytical and analytical procedures that we used were also different. These two technical steps are well known critical factors impacting MP pattern, quantitatively and qualitatively [26]. Despite undertaken standardization attempts [51,52], no consensus for flow-cytometry analysis of MPs has emerged up to now [26] leading to different protocols and doubts on the most accurate strategy to be used. Given the differences between MPs purification procedures, we advocated to report the distributions of FS values for MPs and calibration beads as shown in the S1 Fig. It should be pointed-out that our flow cytometer could not detect small MPs (diameter < 400 nm), which may be relevant bio-markers and bio-effectors. Indeed, we have recently shown that exosomes isolated from SCA patients impacted the endothelial cells phenotype and induced monocyte adhesion in a severity dependent manner [53]. We have also identified signature of exosomal microRNAs that distinguished severe from mild SCA patients [53]. Our present data do not provide evidence that large MPs are biomarkers of the three SCD complications analyzed. Further studies are warranted to better describe the clinical significance of the different sub-types of extracellular vesicles in the sickle cell disease pathophysiological processes by analyzing the quantitative and qualitative patterns of MPs and exosomes in the same patients.

In summary, we have shown that HbSC patients exhibited lower concentration of MPs compared to SCA patients. Furthermore, we provided evidence that RBC-derived MPs exhibited higher density of externalized PS than PLT-derived MPs and thus could be more efficient in triggering coagulation activation. Although we did not detect any relationship between the 3 major SCD complications studied and MP concentrations, further studies are warranted to decipher their roles in the occurrence of SCD clinical manifestations, specifically those associated with a hypercoagulability phenotype.

## Supporting information

S1 Fig Distributions of FS indexes of PLT-derived MPs and RBC-derived MPs in one SCA patient. Size-calibrated beads were used to ensure the reproducibility of FS values determination. Due to the optical properties of FC500 cytometers, 900nm beads have the same FS value than 1μm-large MPs. All MPs detected have a size comprised between 0.4 and 1μm but size distribution for RBC-derived MPs is shifted towards bigger size when compared to PLT-derived MPs.
(TIF)

S1 Table Comparison of biological parameters and clinical expression between HC-treated versus HC-not treated SCA patients. Quantitative variables were summarized as means ± standard deviation or as the median with the interquartile range (IQR) according to their distributions. Intergroup differences were assessed using unpaired t test (*), Mann Whitney test (#) or chi 2 test (&). Significant *p* values are in bold. TRJV: tricuspid regurgitation jet velocity; VOC: vaso-occlusive crisis; ACS: acute chest syndrome.
(TIF)

S2 Table MP concentrations in HC-treated SCA patients and SCA patients not treated with HC. MP concentrations were expressed as median with the interquartile range. Intergroup differences were assessed using the Mann Whitney test. Significant *p* values are in bold. Mono: monocyte; Neutro: neutrophil; Endo: endothelial.
(TIF)
