# Supplementary figures and images for: Differences of microparticle patterns between sickle cell anemia and hemoglobin SC patients
Source: PLoS One. 2017 May 10;12(5):e0177397. doi: 10.1371/journal.pone.0177397 (PMC5425024; doi:10.1371/journal.pone.0177397)

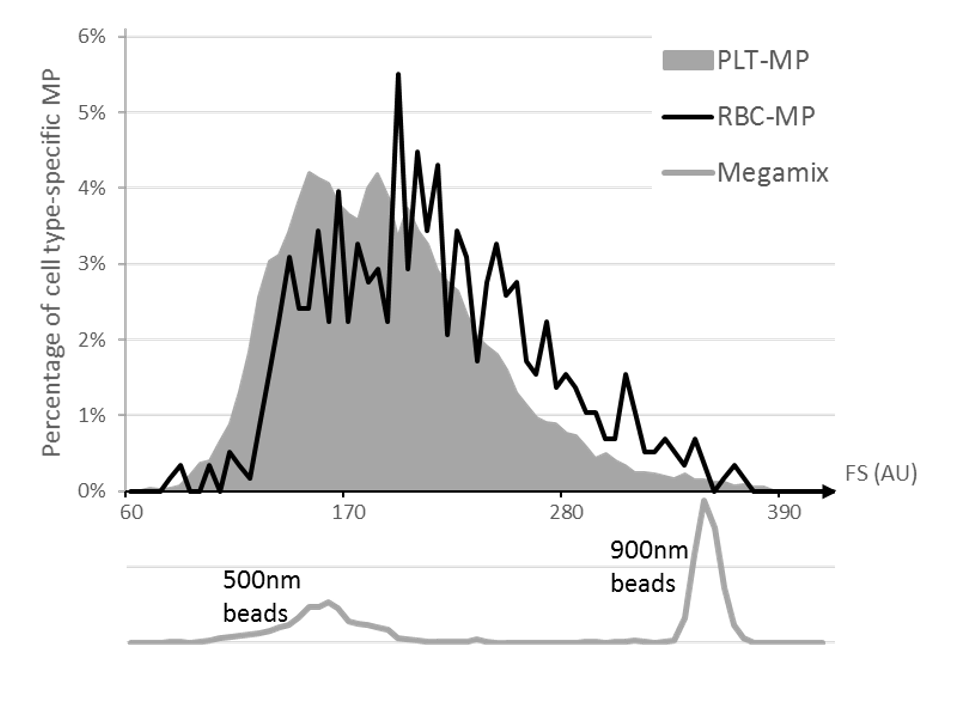

Supplement: S1 Fig — Size-calibrated beads were used to ensure the reproducibility of FS values determination. Due to the optical properties of FC500 cytometers, 900nm beads have the same FS value than 1μm-large MPs. All MPs detected have a size comprised between 0.4 and 1μm but size distribution for RBC-derived MPs is shifted towards bigger size when compared to PLT-derived MPs. (TIF) [file pone.0177397.s001.tif]

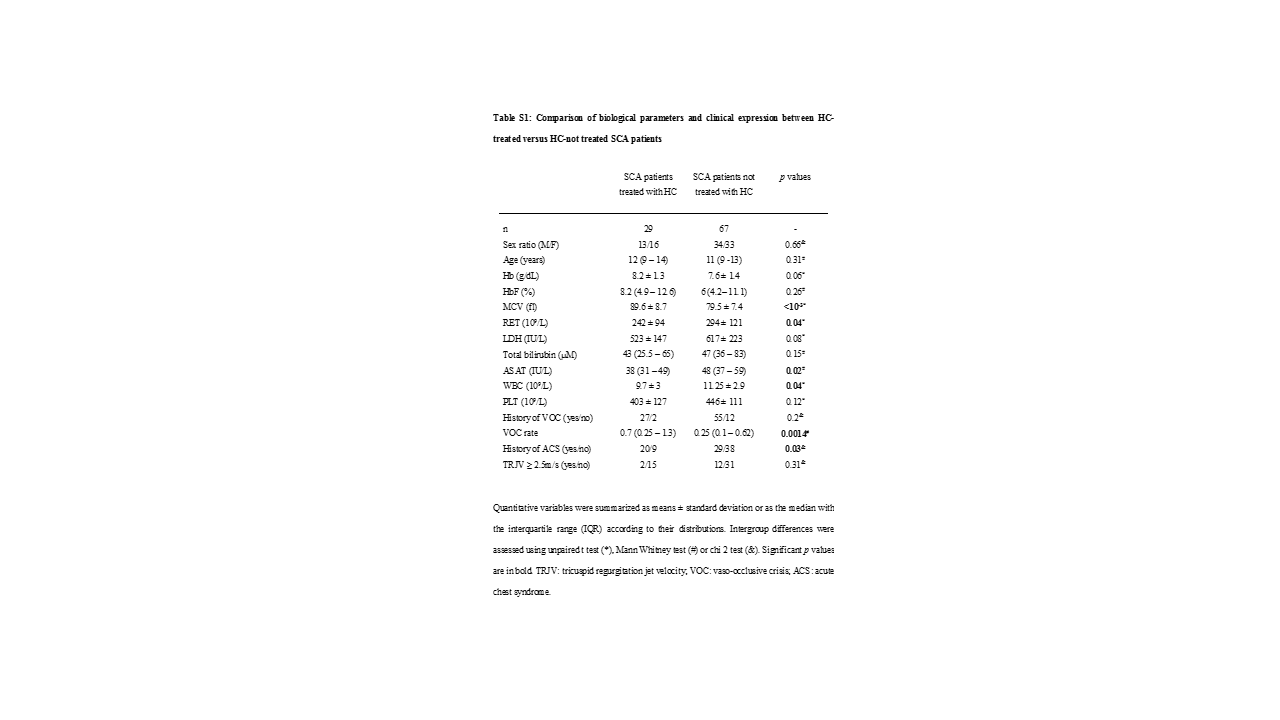

Supplement: S1 Table — Quantitative variables were summarized as means ± standard deviation or as the median with the interquartile range (IQR) according to their distributions. Intergroup differences were assessed using unpaired t test (*), Mann Whitney test (#) or chi 2 test (&). Significant p values are in bold. TRJV: tricuspid regurgitation jet velocity; VOC: vaso-occlusive crisis; ACS: acute chest syndrome. (TIF) [file pone.0177397.s002.tif]

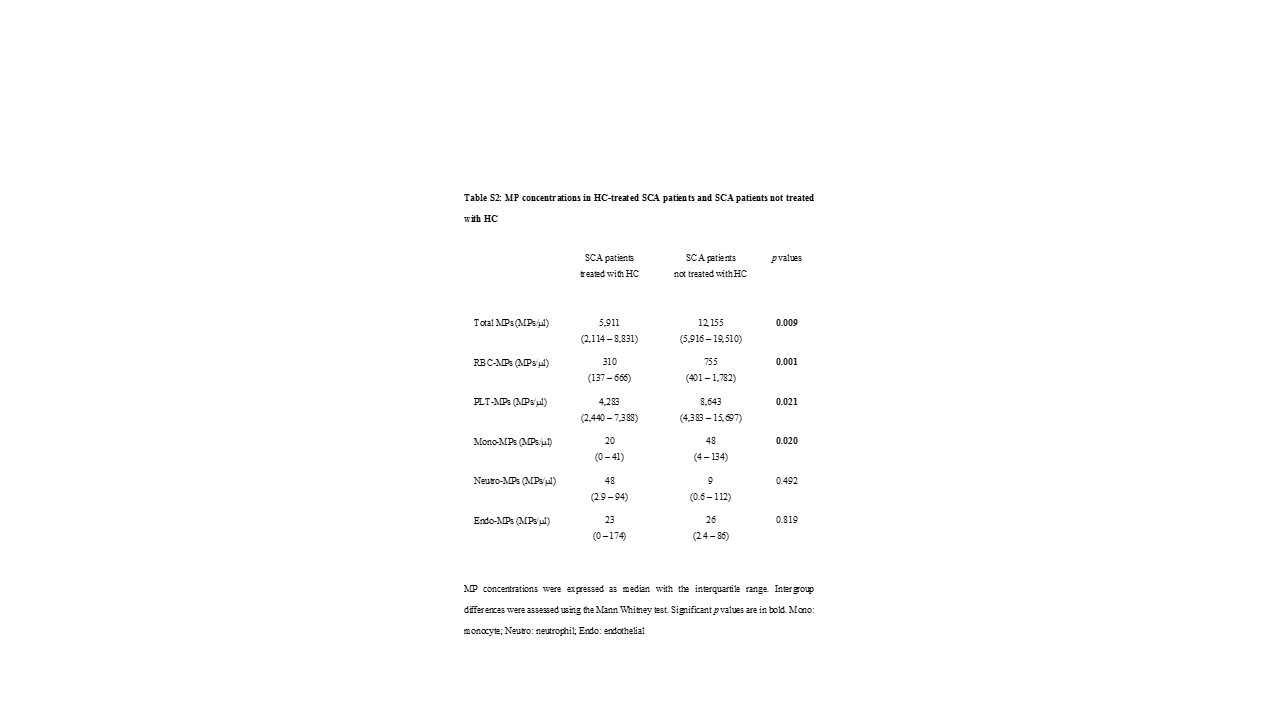

Supplement: S2 Table — MP concentrations were expressed as median with the interquartile range. Intergroup differences were assessed using the Mann Whitney test. Significant p values are in bold. Mono: monocyte; Neutro: neutrophil; Endo: endothelial. (TIF) [file pone.0177397.s003.tif]
